# Efficacy of Phrenic Nerve Block and Suprascapular Nerve Block in Amelioration of Ipsilateral Shoulder Pain after Thoracic Surgery: A Systematic Review and Network Meta-Analysis

**DOI:** 10.3390/medicina59020275

**Published:** 2023-01-31

**Authors:** Tanyong Pipanmekaporn, Prangmalee Leurcharusmee, Yodying Punjasawadwong, Jiraporn Khorana, Artid Samerchua, Wariya Sukhupragarn, Isaraporn Sukuam, Nutchanart Bunchungmongkol, Surasak Saokaew

**Affiliations:** 1 Department of Anesthesiology, Faculty of Medicine, Chiang Mai University, Chiang Mai 50200, Thailand; 2 Clinical Epidemiology and Statistic Center, Faculty of Medicine, Chiang Mai University, Chiang Mai 50200, Thailand; 3 Department of Surgery, Faculty of Medicine, Chiang Mai University, Chiang Mai 50200, Thailand; 4 Maeramat Hospital, Maeramat District, Tak 63140, Thailand; 5 Unit of Excellence on Clinical Outcomes Research and IntegratioN (UNICORN), School of Pharmaceutical Sciences, University of Phayao, Phayao 56000, Thailand; 6 Center of Health Outcomes Research and Therapeutic Safety (Cohorts), School of Pharmaceutical Sciences, University of Phayao, Phayao 56000, Thailand; 7 Division of Social and Administrative Pharmacy, Department of Pharmaceutical Care, School of Pharmaceutical Sciences, University of Phayao, Phayao 56000, Thailand

**Keywords:** ipsilateral shoulder pain, phrenic nerve block, suprascapular nerve block, thoracotomy, video-assisted thoracoscopic surgery

## Abstract

*Background and Objectives*: Ipsilateral shoulder pain (ISP) is a common complication after thoracic surgery. Severe ISP can cause ineffective breathing and impair shoulder mobilization. Both phrenic nerve block (PNB) and suprascapular nerve block (SNB) are anesthetic interventions; however, it remains unclear which intervention is most effective. The purpose of this study was to compare the efficacy and safety of PNB and SNB for the prevention and reduction of the severity of ISP following thoracotomy or video-assisted thoracoscopic surgery. *Materials and methods:* Studies published in PubMed, Embase, Scopus, Web of Science, Ovid Medline, Google Scholar and the Cochrane Library without language restriction were reviewed from the publication’s inception through 30 September 2022. Randomized controlled trials evaluating the comparative efficacy of PNB and SNB on ISP management were selected. A network meta-analysis was applied to estimate pooled risk ratios (RRs) and weighted mean difference (WMD) with 95% confidence intervals (CIs). *Results*: Of 381 records screened, eight studies were eligible. PNB was shown to significantly lower the risk of ISP during the 24 h period after surgery compared to placebo (RR 0.44, 95% CI 0.34 to 0.58) and SNB (RR 0.43, 95% CI 0.29 to 0.64). PNB significantly reduced the severity of ISP during the 24 h period after thoracic surgery (WMD −1.75, 95% CI −3.47 to −0.04), but these effects of PNB were not statistically significantly different from SNB. When compared to placebo, SNB did not significantly reduce the incidence or severity of ISP during the 24 h period after surgery. *Conclusion:* This study suggests that PNB ranks first for prevention and reduction of ISP severity during the first 24 h after thoracic surgery. SNB was considered the worst intervention for ISP management. No evidence indicated that PNB was associated with a significant impairment of postoperative ventilatory status.

## 1. Introduction

Ipsilateral shoulder pain (ISP) is a common complication following thoracic surgery. Its incidence varies between 21% and 97% [1,2,3,4,5,6,7,8,9,10,11,12,13]. The level of ISP is not effectively relieved by thoracic epidural analgesia and systemic opioids [14]. Severe ISP can cause patient dissatisfaction, impaired effective respiration, and postoperative shoulder dysfunction [15]. Therefore, effective and safe intervention for ISP management is imperative. The occurrence of ISP after thoracic surgery relates to a transection of the major bronchus [14], referred pain from an irritation of diaphragmatic pleura, pericardium, and mediastinum via the phrenic nerve [1,5,6,12,13,16], straining of muscles around the shoulder joint during lateral decubitus position [17,18], and pleural irritation from the chest tube [7].

Video-assisted thoracic surgery (VATS) is a minimally invasive surgical procedure that can minimize stress and decrease intensity of postoperative pain compared with thoracotomy. Based on the procedure, specific postoperative pain management (PROSPECT) guidelines, paravertebral block (PVB) with a single shot or continuous infusion of local anesthetic is recommended for incisional pain control after VATS [19], while either PVB or thoracic epidural analgesia (TEA) with local anesthetic and strong opioids is preferable for providing analgesia after thoracotomy. However, the efficacy of phrenic nerve block (PNB) and suprascapular nerve block (SNB) for prevention of ISP could not be demonstrated [20].

Various types of PNB, such as phrenic nerve infiltration (PNI) [1,5,6,12,13,16,21] or PNB under a supraclavicular approach [22], could significantly reduce the incidence and severity of ISP. PNI with long-acting local anesthetics is preferable to lidocaine for relieving ISP up to 24 h after surgery [22]. However, the major concern for PNI with long-acting local anesthetics is possible ventilatory impairment due to prolonged ipsilateral hemidiaphragmatic paresis [7]. When using SNB, the suprascapular nerve directly provides the sensory innervation to the shoulder [9]. Although SNB is an effective analgesic technique for shoulder surgery and chronic shoulder pain [23], its effectiveness for ISP management remains inconclusive. When compared to placebo, preoperative SNB can effectively reduce the severity of ISP after open thoracotomy [24]. PNB is superior to SNB in prevention and control of ISP [6,21]; however, the samples for these studies were small. In addition, various techniques of SNB, including landmark [7], nerve stimulator [9], or ultrasound guidance [19,22], have been performed, and these might preclude the effectiveness of this block. PNB and SNB are anesthetic interventions for ISP management; however, it remains unclear which intervention works better. The network meta-analysis (NMA) is designed to compare different interventions that have never been directly compared with head-to-head randomized controlled trials. NMA simultaneously examines the relative treatment effects of direct and indirect comparisons via a common treatment comparator within a single approach [25] and identifies the best intervention for the outcomes of interest according to their rank [26]. The purpose of this study was to compare the efficacy and safety of PNB and SNB for prevention and reduction of severity of ISP following thoracotomy or VATS.

## 2. Materials and Methods

### 2.1. Literature Search Strategies and Data Extraction

This systematic review was conducted based on the criteria of the Preferred Reporting Items for Systematic Review and Meta-Analysis (PRISMA) [27] with Extension Statement for Reporting of Systematic Reviews Incorporating Network Meta-Analyses of Health Care Interventions. The registration code with the International Prospective Register of Systematic Reviews (PROSPERO) was CRD42020204473 [28]. The original articles were systematically searched without language restriction from PubMed, Web of Science, Embase, Ovid Medline, Science Direct, Scopus, Cochrane Central Register of Controlled Trials (CENTRAL) and Cochrane Library, and Google Scholar from the inception of each publication to 30 September 2022. Studies to be included were the randomized controlled trials that assessed the effectiveness of PNB or SNB for the prevention of ISP following open thoracotomy or thoracoscopy for noncardiac surgery. Studies that were not original articles, such as editorials, letters, case reports, case series, observational studies, and meta-analyses, were excluded. The search-term algorithm was modified for each database with a combination of relevant terms according to Cochrane for systematic reviews of randomized controlled trials (RCTs) [29]. Various combinations, vocabulary terms, keywords, and medical subject headings (MESH) about ISP were sought. The search terms included ipsilateral, shoulder pain, and thorac* surgery. The results were combined with the search terms PNB, SNB, AND open thoracotomy or thoracoscopy. Reference lists for relevant studies were screened. The details of search strategies are described in the Supplementary Materials (Table S1).

### 2.2. Inclusion Criteria

RCTs that included the use of PNB or SNB to prevent the occurrence of ISP and decrease ISP severity in adults aged 18 years old or older in patients undergoing open thoracotomy or VATS for noncardiac surgery were included. The details of intervention and cointervention of included studies are described.

### 2.3. Study Outcomes

The primary outcome of interest was the incidence of ISP. This study measured this outcome as the proportion of patients who developed a new onset of ISP during the 24 h period following thoracic surgery. The secondary outcomes were degree of shoulder pain severity during the first 24 h period following surgery and any adverse events related to the interventions. The measured pain scales of ISP were converted to numeric rating scale, visual analogue scale (VAS) or verbal ranking score (VRS) of 0 to 10 during the 24 h after the operation (i.e., effect on reduction of the incidence and severity of ISP). Interventions were categorized as (1) better than the reference intervention (placebo) and also superior to the other intervention at the same time—“best interventions”, (2) no difference from placebo—“worst intervention”, (3) superior to placebo, but not superior to other interventions—“inferior to the best, but better than the worst intervention” [26]. Adverse events related to the interventions, such as postoperative ventilatory status, respiratory function impairment, and upper-extremity weakness were assessed. Description of intervention and cointervention (rescue medication and intraoperative positioning) of included studies in network analysis are presented in the Supplementary Materials (Table S2).

### 2.4. Data Extraction and Data Retrieval

Study selection was conducted in the following steps. Three independent investigators (TP, PL, IS) reviewed the title and abstracts. Thereafter, the full articles were retrieved by literature search according to the predefined searching algorithms if a decision could not be made following the initial phase. Disagreements were resolved by discussion with the third investigator (YP). RCTs were enrolled if they met the predefined inclusion criteria. Two reviewers (TP, PL) independently collected relevant data from each included study using the standardized data extraction form. Data extracted from studies comprised study characteristics, year of publication, characteristics of patients and surgical procedures, types of intervention and comparators, incidence and severity of ISP, rescue treatment for ISP, and adverse effects related to the interventions. If the reported information were incomplete, the authors would contact the study investigators by email.

### 2.5. Quality Assessment

The enrolled studies were independently assessed by two reviewers (WS, AS) using the Jadad score [30] and Cochrane Collaboration Risk of Bias Tools (RoB) [27]. The maximum Jadad score is 5 points, with studies having a total score equal or greater than 3 defined as high quality [31]. The RoB evaluates bias in intervention studies and consists of 6 domains: sequence generation, allocation concealment, blinding of participants and personnel, blinding of outcome assessment, incomplete outcome data, selecting outcome report, and other source of bias. Each domain was rated “yes”, “no”, or “unclear” for low, high, and unclear risk of bias. If the first three criteria were answered with “yes” and there was no concern with the last three questions, then the study was classified as “low risk of bias.” Studies with ≤2 domains being “unclear” or “no” were classified “moderate risk of bias.” Studies with ≥3 domains being “unclear” or “no” were classified “high risk of bias” [31]. Any disagreements between two reviewers were resolved by discussion to find a consensus.

### 2.6. Statistical Analysis

The main characteristics are summarized using descriptive statistics. Categorical variables are summarized using numbers and proportions, and continuous variables are reported using means and standard deviation (SD). If no data were available, the means and SD were estimated from the median and interquartile range (IQR), as described by Hozo et al. [32]. A pairwise meta-analysis with a random-effect model was used to estimate treatment effects, pool risk ratios (RRs) and weighted mean difference (WMD) with 95% confidence intervals (CIs) for binary and continuous outcomes, respectively. Heterogeneity in pairwise meta-analysis for all direct comparisons was assessed using χ^2^ and I^2^ tests. I^2^ > 50% was considerable statistical heterogeneity.

A random-effect network meta-analysis was used to indirectly compare intervention effects for all interventions in the following steps. Coefficients (i.e., lnRR) along with variance–covariance of comparisons were estimated for each study using placebo or common interventions as comparators. Then, these lnRRs were pooled across studies using a multivariate meta-analysis with restricted maximum likelihood function. For multiarm studies, the side-splitting model was used to estimate parameters for both sides. We calculated the indirect estimate as the differences for the direct estimates and obtained 95% predictive intervals (PrI) by normal approximation. Ninety-five percent PrIs were reported to assess their uncertainty and magnitude of heterogeneity in the NMA. Predictive intervals provided an interval in which future studies will fall. Network inconsistency assumption was disagreement between direct and indirect estimates (also called inconsistency factor (IF)), which was assessed by design-by-treatment and node-splitting technique models. Consistency–inconsistency was analyzed to estimate inconsistency globally and the node-splitting technique was used to evaluate inconsistency locally with all closed loops. The IF with corresponding 95% CI was analyzed in each triangular or quadratic loop. The IF was tested using Z test. The model is considered inconsistent if the IF is different from 0 (*p* > 0.05) which indicates no evidence of inconsistency. We then assessed the common network heterogeneity using tau-squared (tau^2^), I^2^ and the 95% CI of I^2^. The surface under the cumulative ranking curve (SUCRA) was analyzed based on a Bayesian approach to measure the ranking and the uncertainty. The probability of being the best intervention was evaluated.

An adjusted funnel plot was built to determine small study effects. Sensitivity and subgroup analysis were analyzed based on size of included RCTs. We performed sensitivity/subgroup analysis according to type of surgical approach (open thoracotomy or VATS), period of study (before 2010 and 2010 and after), and high-quality RCTs (Jadad score less than 3). All analyses were performed using Stata 15.0 (StataCorp, College Station, TX, USA). A *p* value < 0.05 was considered statistically significant.

## 3. Results

**Figure 1:**
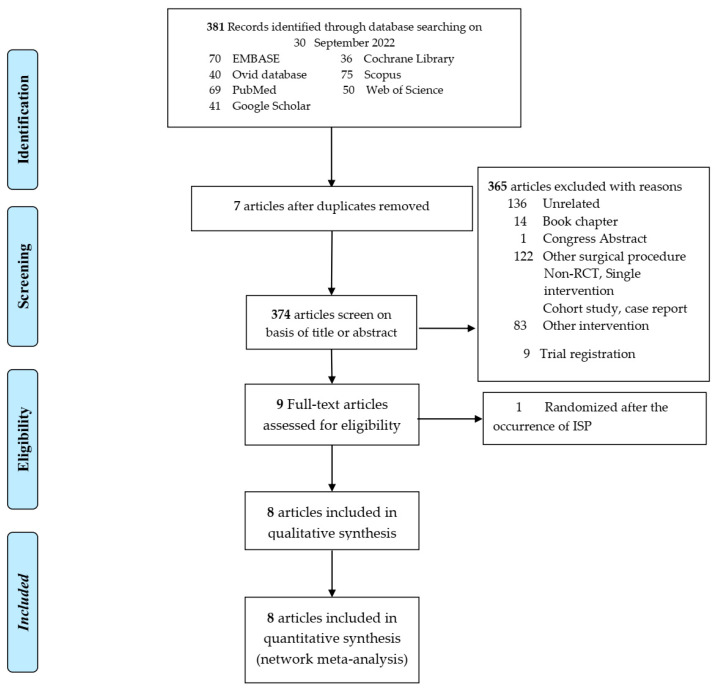
PRISMA flow diagram of selected articles.

A total of 381 records were identified. After removal of duplicate studies, 374 articles were screened on basis of title and abstract. In sum, 365 articles were excluded and 8 retrieved for review, with the reasons shown in Figure 1.

Seven RCTs were two-arm trials and one was a three-arm trial. Among seven trials investigating the effect of PNB, six trials (87.5%) were the PNI and the other trial was supraclavicular approach of the PNB. The details of interventions and cointerventions of enrolled studies are described in the Supplementary Materials (Table S3).

### 3.1. Characteristics and Quality of Included Studies

**Figure 2:**
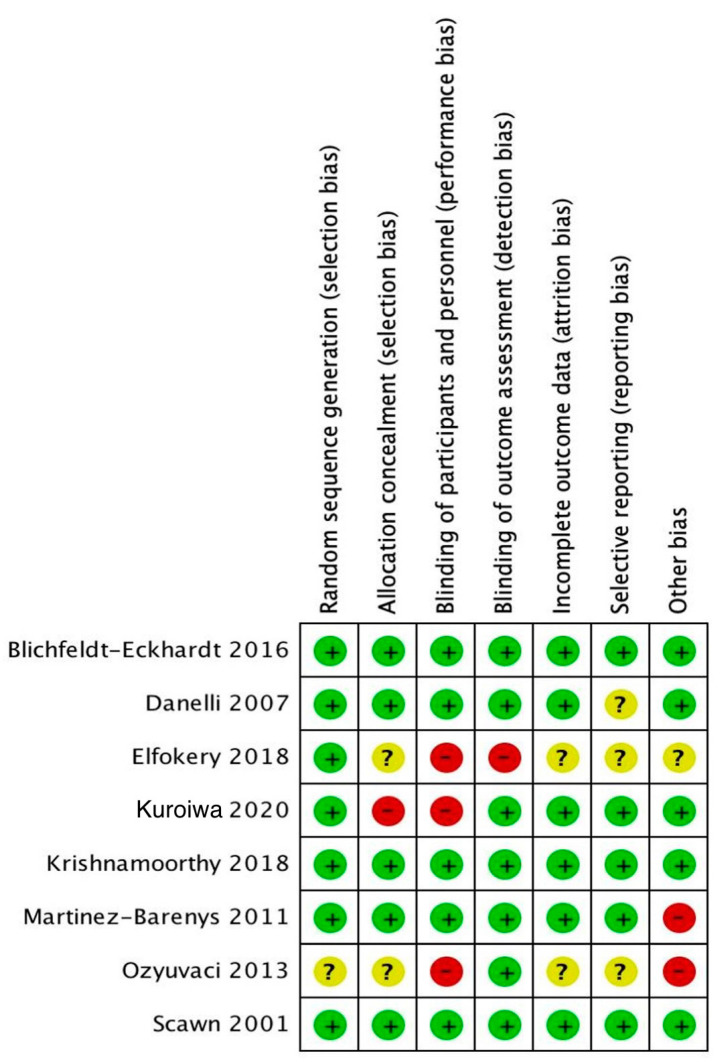
Risk of bias of all included studies. (green for low risk of bias; yellow for unclear risk of bias; –red for high risk of bias) [1,5,6,16,21,22,24,33].

**Figure 3:**
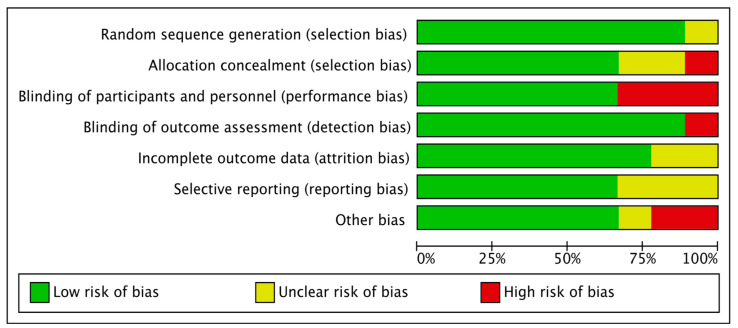
Overall risk of bias in included studies: review authors’ judgment (low, unclear, high) for risk-of-bias item shown as percentages across all included studies.

The characteristics of included studies are described in Table 1. The eight studies included contained 587 participants (range of 36 to 90 participants). The median age of study populations was 63.2 years (IQR 54.5–67.7%) and 44.4% of patients were male (IQR 35.5–73.3%). A majority of surgical approach and surgical procedures were open thoracotomy (six of eight studies, 75%) and lobectomy (five of eight studies, 62%), respectively. The methodological quality of the eight RCTs included in the systematic review was high, as shown by Jadad scores of 3 or above (scale range of 0–5), except the study of Ozyuvaci et al. [24], which had a Jadad score of 2. The individual and overall risk of bias for the included RCTs are shown in Figure 2 and Figure 3. Three studies had a high risk of bias. An appropriate allocation concealment process was properly performed in five studies. In the blinding participants and personnel process, five studies were low risk and three were high risk of bias (37.5%). Blinding outcomes assessment was clearly described in seven of the eight studies.

### 3.2. Meta-Analysis and Network Meta-Analysis

#### 3.2.1. Incidence of ISP (Network Meta-Analysis)

**Figure 4:**
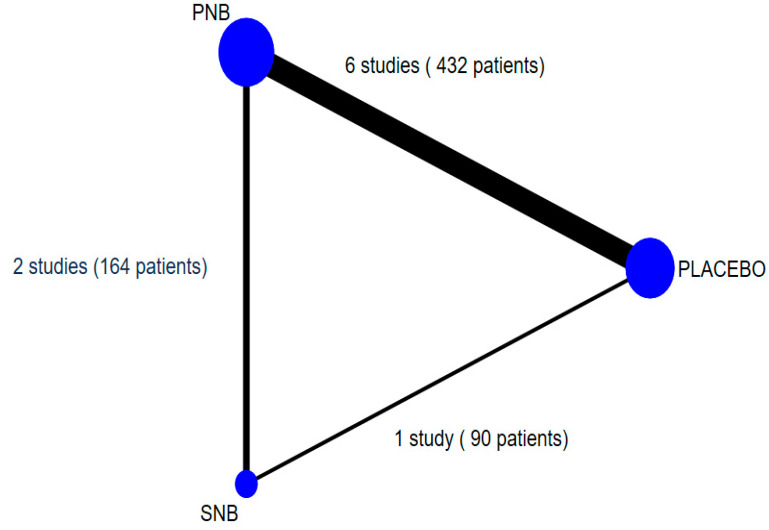
Network map of included studies for the incidence of ISP. Width of the lines is the number of trials comparing pairwise intervention. Size of the circles is proportion of the sample. ISP, ipsilateral shoulder pain.

A total of 551 patients from seven studies were available for analysis of the incidence of ISP [1,5,14,22,33], one study comparing PNB to SNB [6], and one multiarm study comparing PNB and SNB to placebo [21]. One study comparing SNB and placebo [24] was excluded due to no data on the incidence of ISP (Figure 4). The results of NMA and pairwise meta-analyses for the incidence of ISP during 24 h after thoracic surgery are summarized in Table 2. PNB significantly reduced the incidence of ISP during 24 h after operation when compared to placebo (RR 0.44, 95% CI 0.34 to 0.58) and SNB (RR 0.43, 95% CI 0.29 to 0.64).

**Figure 5:**
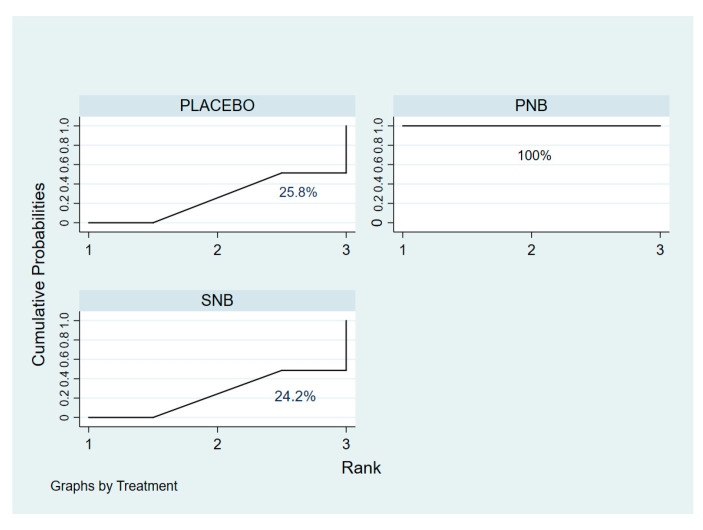
SUCRA ranking curves for prevention of ISP during the first 24 h after surgery. SUCRA, surface under the cumulative ranking curve; ISP, ipsilateral shoulder pain.

**Figure 6:**
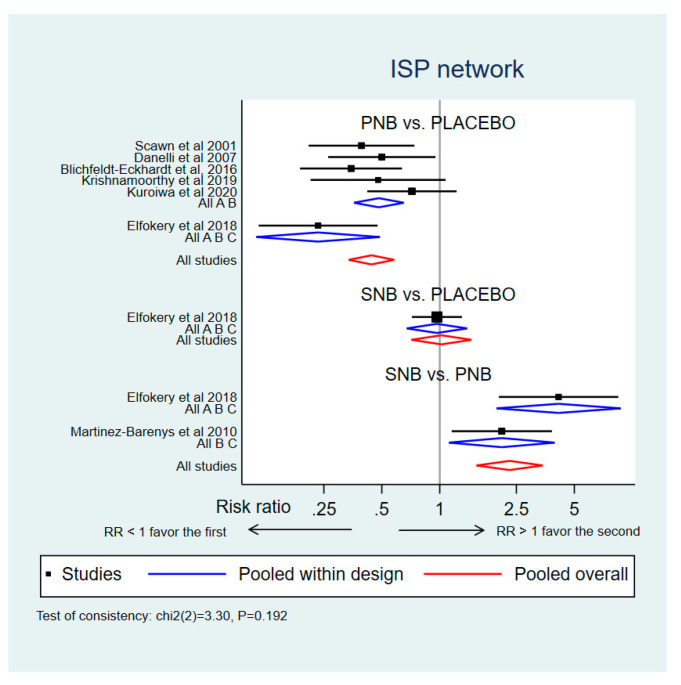
Network meta-analysis results for ISP prevention during 24 h after surgery. PNB is associated with lower risk of ISP than placebo and SNB. Forest plot: RR < 1 indicated that the first treatment in pairwise comparison is associated with lower risks of ISP; thus, the first treatment was favored compared to the second. While RR > 1 indicated that the first treatment in pairwise comparison is associated with increased risks of ISP; the second treatment was favored compared to the first. ISP, ipsilateral shoulder pain; RR, risk ratio; PNB, phrenic nerve block; SNB, suprascapular nerve block [1,5,16,21,22,33].

Network meta-analysis showed that ISP incidence at 24 h after surgery for SNB and placebo was not significantly different. There was no evidence of inconsistency during the global test (χ^2^ statistic = 3.30; *p* = 0.192) or loop inconsistency (IF 0.33, 95% CI 0.00 to 1.03, tau^2^ = 0.015; *p* = 0.345) (Supplementary Materials Tables S4 and S5). This is indicated by the largest SUCRA of prevention of ISP in the PNB (Figure 5). PNB was associated with the lowest risk of ISP compared to placebo and SNB (Figure 6). The predictive interval plot shows that PNB prevents the risk of ISP compared to placebo and SNB, whereas the effect of SNB and placebo for prevention of ISP were comparable (the Supplementary Materials Figure S1). PNB significantly decreased the incidence of ISP compared to placebo (RR 0.50, 95% CI 0.30 to 0.85) and SNB (RR 0.41, 95% CI 0.19 to 0.87) at the first 48 h after surgery. Risk of ISP between SNB and placebo at 48 h was comparable (RR 1.23, 95% CI 0.49 to 3.07).

#### 3.2.2. ISP Severity (Network Meta-Analysis)

A total of 537 patients from seven studies were available for analysis of ISP severity. There were five studies comparing PNB versus placebo or no block [1,14,20,22,33], one study comparing PNB versus SNB [6], and one multiarm study comparing PNB, SNB and placebo [21] during the first 24 h after surgery (Supplementary Materials Figure S2). The global test for inconsistency and loop-specific heterogeneity suggested the presence of inconsistency (χ^2^ statistic = 42.65; *p* < 0.001 for global test and IF 3.97, 95% CI 1.10 to 6.84, tau^2^ = 1.579, *p* = 0.007 for loop test) (Supplementary Materials Table S5). Patients receiving PNB had significant reduction in ISP severity compared to placebo (WMD −1.75, 95% CI −3.47 to −0.04) (Table 3). PNB significantly reduced the severity of ISP compared to placebo (WMD −1.60, 95% CI −2.97 to −0.22) at 36 h. The severity of ISP in SNB was comparable with that in PNB and placebo at 6, 24 and 36 h, respectively.

**Figure 7:**
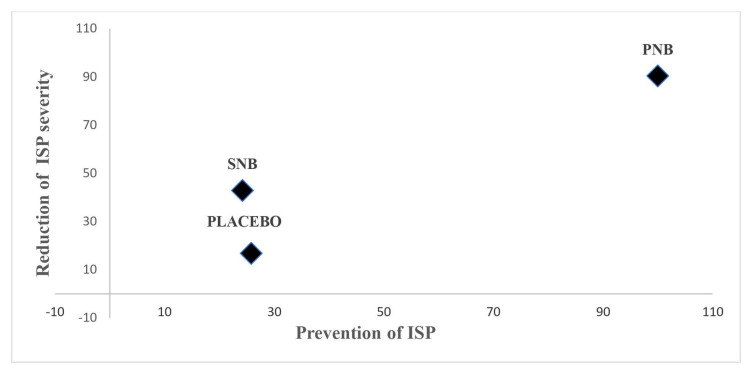
Ranking plot for ipsilateral shoulder pain network during 24 h postoperative period. Treatments have been ranked according to the surface under cumulative ranking curves (SUCRA) between the effective treatment of the incidence and severity of ISP. Each plot was located according to their SUCRA values of each anesthetic intervention for two outcomes. The larger SUCRA value mean a better ranking of the intervention. PNB has the highest SUCRA value and is regarded as the most preferred intervention for prevention and reduction of ISP severity. SUCRA, the surface under cumulative ranking curves; ISP, ipsilateral shoulder pain; PNB, phrenic nerve block; SNB, suprascapular nerve block.

Network meta-analysis showed that degree of ISP severity between SNB and placebo for 24 h was comparable. Network ranking of cumulative probability indicated that PNB had the largest SUCRA for reduction in ISP severity, meaning that PNB was the best treatment (Supplementary Materials Figure S3). Network ranking of cumulative probability suggested that PNB was the best treatment followed by SNB and placebo, respectively (Figure 7). Network meta-analysis revealed significant lower mean differences of ISP severity for PNB than placebo, whereas degree of ISP severity between PNB vs. SNB and SNB vs. placebo were not significantly different (Supplementary Materials Figures S4 and S5).

#### 3.2.3. Postoperative Ventilatory Status Indicated by PaCO_2_ (Meta-Analysis)

A total of 167 patients from three studies comparing PNB to control were available for analysis. There was not enough direct evidence to analyze network meta-analysis; therefore, meta-analysis with random effect was performed for pairwise comparisons (Supplementary Materials Table S6). No significant difference in postoperative CO_2_ level between PNB and placebo was detected, with evidence of considerably heterogeneity (WMD 0.25; 95% CI −2.71 to 3.20, *p* = 0.869; I^2^ = 75.9% for heterogeneity) (Supplementary Materials Table S6).

#### 3.2.4. Sensitivity Analysis and Publication Bias

Sensitivity analysis for the incidence and severity of ISP was conducted to evaluate the robustness of the following results from the main analysis, analyzed according to the incidence and severity of ISP at 6 h after surgery; 24 h after surgical procedure (excluding Scawn et al. and Blichfeldt-Eckhardt et al. for providing the ISP incidence only at 6 h); thoracotomy (excluding Blichfeldt-Eckhardt et al. and Kuroiwa et al. for VATS); studies conducted since 2010 (excluding Scawn et al. and Danelli et al.) and high-quality RCTs (excluding Ozyuvaci et al. (Jadad score < 3 points)). The results of sensitivity analysis for the incidence of ISP remained consistent with the main analysis for all interventions and there was no evidence of inconsistency (Supplementary Materials Table S7), whereas the results of sensitivity analysis for the severity of ISP indicated consistency only in studies focusing on ISP during the first 6 h after surgery (Supplementary Materials Table S8). PNB significantly reduced mean pain scores for ISPs compared to placebo during the first 6 h after surgery (WMD −2.02, 95% CI −3.21 to −0.84) and 24 h (WMD −2.27, 95% CI −4.05 to −0.48). Comparison-adjusted funnel plots of all pairwise comparisons for the incidence and severity of ISP showed some degree of asymmetry, which indicated potential publication bias (Supplementary Materials Figures S6 and S7).

## 4. Discussion

This systematic review and NMA compared the relative effects of PNB and SNB in terms of prevention and reduction of ISP after thoracic surgery. The network pool outcome estimates revealed a significant reduction in the incidence of ISP during the first 24 h that favored PNB when compared with SNB and placebo. Primarily, the severity of ISP at 24 h after PNB was significantly lower than placebo, but not different from SNB. The incidence and severity of ISP for SNB were not significantly different from placebo. These findings were also consistent during sensitivity and subgroup analysis.

Two main hypotheses have been proposed for the occurrence of ISP. Firstly, the pain is transmitted from an irritated pericardium, mediastinum as well as diaphragmatic pleura caused by the surgical procedure [1] and chest tube [7] via the phrenic nerve. This hypothesis was strongly supported by the findings of several studies demonstrating the effectiveness of PNB [1,5,6,12,13,16,21,22] incidence and severity of ISP. Furthermore, this etiology was strongly supported by the occurrence of ISP induced by the phrenic nerve stimulation device [34]. Secondly, other causes of ISP include ligament distraction and shoulder injury during rib spreading and scapula retraction [17,18]. Prolonged duration of surgery and improper positioning of patients could increase risk of ISP by stressing on the rotator cuff muscle [35]. The suprascapular nerve, which has the same origin as the phrenic nerve, provides a major sensory innervation to the shoulder and also transmits somatic pain caused by an excessive strain of ligament and shoulder during the surgical procedure or improper positioning [9]. The suprascapular nerve also conveys motor innervation of infraspinatus and supraspinatus muscle and supplies sensation of posterior aspect of rotator cuff except infraspinatus and supraspinatus muscle. Therefore, SNB may be ineffective for relieving shoulder pain originating from infraspinatus and supraspinatus muscles after thoracic surgery [35]. Other etiologies of ISP included transection of major bronchus [16], myofascial involvement [35], and pleural irritation from the chest tube [7]. Management of ISP should be based on the possible etiology. Consequently, PNB and SNB are analgesic modalities of interest.

Techniques of anesthetic intervention also have an influence on the incidence, severity of ISP and adverse effects to the block. PNB mainly focuses on management of the visceral component of ISP or referred ISP [36], while SNB aims to alleviate the somatic component or musculoskeletal ISP [8].

In this NMA, a majority of PNB techniques were PNI near the level of the diaphragm and required periphrenic fat pad as the reservoir for delivering local anesthetics [6]. The advantages of PNI include reduced risk of nerve damage from direct intraneural injection [16], increased the possibility of phrenic nerve confirmation during the intraoperative period [33], and reduced upper-extremity weakness [22]. The results of several studies supported PNI being able to significantly reduce the incidence and severity of ISP compared to SNB [6,21] and placebo [1,5,16,21]. Corresponding to the findings of a recent meta-analysis focusing on the effectiveness of PNI and ISP [37], PNI significantly decreased the incidence and severity of ISP compared to placebo during 6 and 24 h after thoracotomy, whereas a previous study found that the use of PNI at the level of the azygous vein significantly reduced the severity but not the incidence of ISP compared to placebo during 24 h after VATS [33]. Some sensory fibers leave the phrenic nerve to supply parietal and mediastinal pleura earlier than the location of PNI leading to inadequate PNB. Therefore, the proximal approach of PNB could provide superior analgesia. This was supported by an effective management of ISP with supraclavicular approach of PNB compared to placebo for major thoracic surgery [22]. In addition, this study found that SNB was the worst intervention for ISP management. Corresponding to the findings of a few RCTs, SNB was worse than PNB and placebo for reduction of the incidence and severity of ISP during 48 and 72 h after open thoracotomy [6,21]. Our study did not include the study of Tan et al. [9], which investigated the effect of SNB on the severity of ISP, because they began randomization after patients had experienced ISP at a postanesthesia care unit. There were some possible explanations regarding the effect of SNB on the incidence and severity of ISP. Firstly, ISP was mainly associated with referred pain through the phrenic nerve rather than suprascapular nerve. Secondly, various techniques of SNB and time to perform SNB, such as before [21,24] or after the operation [6], had been used among studies that should be considered to determine the effectiveness of SNB for the occurrence of ISP. Finally, most of the included studies regarding SNB showed considerable risk of bias. Further well-designed RCTs with a large samples are required to address the potential effects of SNB on the incidence and severity of ISP after thoracic surgery.

Previous studies reported that nonsteroidal anti-inflammatory drugs (NSAIDs) might be effective for treatment ISP [1,4,14]. Our previous study demonstrated that intravenous parecoxib, cyclooxygenase (COX)-2-selective inhibitor could significantly decrease the incidence and severity of ISP [10]. Conventional NSAIDs, including ketorolac [1,21], ibuprofen [22], and metamizole [6], were used as rescue treatment for ISP in the included studies. This might have an impact on the efficacy of PNB or SNB. The authors further determined the effectiveness of PNB and SNB according to the use of NSAIDs for treatment of ISP. Among four studies [1,6,21,22] using NSAIDS as a rescue treatment for ISP, we found that PNB significantly reduced the incidence (RR 0.37, 95% CI 0.23 to 0.59) and severity of ISP at 6 h compared to placebo (WMD −2.22, 95% CI −3.43 to −1.01),while PNB significantly decreased the incidence (RR 0.44, 95% CI 0.27 to 0.73) and severity of ISP (WMD −2.04, 95% CI −3.37 to −0.70) compared to SNB at 6 h and the risk of ISP for SNB was comparable to placebo (RR 0.84, 95% CI 0.50 to 1.38). The other four studies [5,14,24,33] did not use NSAIDS as the rescue treatment for ISP. The authors found that PNB significantly decreased the incidence of ISP (RR 0.54, 95% CI 0.37 to 0.78) but did not reduce the severity of ISP at 6 h (WMD −1.82, 95% CI −3.69 to 0.05) compared to placebo, whereas PNB did not reduce the severity of ISP (WMD −0.26, 95% CI −3.15 to 2.64) compared to SNB (not reported in the study), and the effectiveness of SNB on the severity of ISP was comparable to placebo in the same period (WMD −1.56, 95% CI −3.77 to 0.65). However, there was an inconsistency in global and nod-splitting techniques in both models during an analysis. Therefore, further studies are required to determine the effect of NSAIDs on the incidence and severity of ISP.

The relationship between type of surgical approach and ISP is controversial. A few studies concluded that type of surgical approach was not associated with the incidence of ISP [35,37]. Whereas a previous cohort study reported that the incidence of ISP in thoracotomies patients (58.5%) was higher than VATS (20.9%) and thoracotomy was an independent risk factor of ISP [16]. However, this might be probably that thoracotomy was applied in major pulmonary resection, while majority of VATS was used for minor thoracic procedure. Furthermore, type of surgical approaches was related to the effectiveness of PNI. This was supported by the finding of a previous meta-analysis which demonstrated that PNI could significantly lower the severity of ISP at 6 h in VATS compared to thoracotomies [37]. As mentioned earlier, type of surgical approaches has an impact on the incidence and severity of ISP as well as the effectiveness of PNB and should be taken into considered during the analysis. However, subgroup analysis according to the type of surgical approach could not be performed due to a small number of trials with VATS.

The effective pain management strategy after thoracic surgery is necessary in order to help patients be able to differentiate the incisional pain from ISP. In this NMA, TEA was selected as the main anesthetic technique for incisional pain control after thoracotomy or VATS (7 of 8 studies), while the other study used thoracic paravertebral block (TPVB) instead [16]. In addition to TEA and TPVB, the use of multimodal analgesia including conventional NSAIDs [1,6], intravenous paracetamol [5,22] and strong opioids [16,22,33] were given. These following standardized protocols for pain management after thoracotomy of all included studies are corresponded with the recommendation of PROSPECT guidelines [20]. However, TEA is not recommended for VATS because of its invasiveness and risk of hypotension, urinary retention and so on [19]. In addition, SNB is not recommended for treatment of ISP after thoracotomy according to the PROSPECT guidelines due to lack of published evidences [20].

Adverse effects are one of the main considerations and should be weighed up between risks and benefits for each intervention. The main disadvantages of PNB include phrenic nerve paralysis, which could have a deleterious effect on the ventilation. However, due to limitation of data available regarding adverse events related to interventions of included studies, we could only perform pairwise comparisons for postoperative PaCO_2_ level in particular PNB studies. There was no significant difference in postoperative PaCO_2_ between PNB and placebo [1,5] or PNB and SNB [22]. Two studies reported no significant difference in PaCO_2_ level between PNB and placebo [21,22]. However, neither provided specific levels of PaCO_2_, and thus could not be included during the pairwise meta-analysis. The values of PaCO_2_ were not present in the other two studies [16,33]. Three of seven studies for PNB reported postoperative respiratory function parameters. Blichfeldt et al. reported no significant difference in forced expiratory volume and forced vital capacity between PNB and placebo [22]. The other two studies demonstrated that PNB produced a significantly lower peak expiratory flow rate [21] and lower peak flow measurement compared to placebo, but no serious complications were observed [16]. Although PNI can cause transient diaphragmatic paralysis, this study could not assess the risk of PNI on postoperative respiratory function based on the current evidence. Upper-extremity weakness was one of the adverse events related to PNB or SNB. In this analysis, only two studies concluded that PNB did not significantly increase in the incidence of upper-extremity paralysis [22] or reduce the range of shoulder movement compared to placebo [33].

This study has some strengths. This is the first study to systematically review using NMA to determine the best anesthetic intervention between PNB and SNB for prevention and reduction severity of ISP following noncardiac thoracic surgery. Contrary to standard meta-analysis, NMA helped us to better understand the relative effects of treatment comparators with the absence of head-to-head comparative trials. Treatment effects are analyzed for a network of treatment based on direct and indirect evidence. This finding could provide more information for anesthesiologists to make their decision on the best interventions for prevention and reduction severity of ISP in order to improve and enhance recovery after thoracic surgery. There are also some limitations in this study. Firstly, there are some heterogeneities from outcome of interventions, various period of follow-up time, including short (6 h) and long (48–72 h), among studies and period of study. Secondly, some of the studies were rated as unclear or had high risk of bias. Allocation concealment and blinding of study group were not achieved. Therefore, the quality of included studies may have an influence on the quality of NMA. Thirdly, numbers of enrolled RCTs and subjects were relatively small, possibly affecting the precision of estimated treatment effect. Fourthly, although interscalene block (ISB) was also an effective anesthetic intervention for prevention of ISP particularly musculoskeletal ISP [3,18,38,39], this intervention could not be included in this analysis due to the limited number of ISB studies available. Fifthly, the result of this study may not be generalized to patients with preexisting shoulder pain before surgery which was the common exclusion criteria for most of included studies. Next, if peridural anesthesia, paravertebral blockades, postoperative opioids, NSAIDs are or are not used during perioperative period, this may have had an impact on the effectiveness of PNB or SNB on the incidence of ISP. A previous cohort study reported that the use of TEA higher than the fifth thoracic vertebrae (T5) could reduce the incidence of ISP [40]. As mentioned above, NSAIDs could significantly reduce the incidence and severity of ISP.

Further study is required to draw the final conclusion. Finally, various adverse effects related to interventions including respiratory function measurement, numbers of patients with pulmonary complications and upper motor weakness among studies were not consistent and could not be analyzed due to limitations of the data available in enrolled studies.

## 5. Conclusions

This systematic review and network meta-analysis demonstrated that PNB ranked first over SNB and placebo for prevention and reduction of ISP severity during the first 24 h after thoracic surgery. SNB was considered the worst intervention for ISP management. PNB might not significantly impair postoperative ventilatory status. Additional studies with large samples are needed to address the potential effects of SNB and adverse events related to individual anesthetic interventions.

## Figures and Tables

**Table 1:** Description of included studies.

Author, Period, Country	Surgical Approach (%)	Jadad Scale	Surgical Procedure (%)	Outcomes	Intervention: Study Size Injection Site, Injectate		Incidence of ISP (%) (Treat vs. Comp)	Age of Patients (Years)	
					Treat	Comp		Treat	Comp
Scawn et al. 2001 [1], United Kingdom	Thoracotomy	5	Pneumonectomy (24.5) Lobectomy (61) Wedge resection (12), Inoperable (2.5)	Incidence Severity of ISP Safety of PNB - PaCO_2_	PNB: (*n* = 21) Periphrenic fat pad at level of diaphragm, Lidocaine	PLB: (*n* = 20) Periphrenic fat pad at level of diaphragm, 0.9% saline	33.3 vs. 85 (6 h)	PNB: 64 (61–67)	PLB: 67 (63–68)
Danelli et al. 2007 [5], Italy	Thoracotomy	5	NR	Incidence Severity of ISP Safety of PNB - PaCO_2_ - PaO_2_/FiO_2_	PNB: (*n* = 25) Periphrenic fat pad at level of diaphragm, Ropivacaine	PLB: (*n* = 25) Periphrenic fat pad at level of diaphragm, 0.9% saline solution	32 vs. 64 (24 h)	PNB: 65 (32–77)	PLB: 66 (41–77)
Martinez Barenys et al. 2011 [6], Spain	Thoracotomy	5	Major resection (87), Sublobar resection (13)	Incidence Severity of ISP	PNB: (*n* = 37) Periphrenic fat pad 1–2 cm proximal at level of diaphragm, Lidocaine	SNB: (*n* = 37) Supraclavicular fossa (after chest closure), Bupivacaine	27 vs. 56.7 (24 h)	PNB: 62.8 ± 10.5	SNB: 63.2 ± 12.7
Ozyuvaci et al. 2013 [24], Turkey	Thoracotomy	2	NR	Severity of ISP	SNB: (*n* = 18) Ultrasound guided, Levobupivacaine	NO (*n* = 18)	NR	SNB: 61.8 ± 8.7	NO: 57.5 ± 8.2
Blichfeldt-Eckhardt et al. 2016 [22], Denmark	Thoracotomy (72.4), VATS (27.6)	5	Pneumonectomy (9.5), Lobectomy (90.5)	Incidence Severity of ISP Safety of PNB - FEV1 - FVC - PaCO_2_ - Incidence of upper motor limb block	PNB: (*n* = 38) Ultrasound-guided supraclavicular fossa (after chest closure), Ropivacaine	PLB: (*n* = 38) Ultrasound-guided supraclavicular fossa (after chest closure), 0.9% saline solution	23.7 vs. 68 (6 h)	PNB: 68.1 ± 8.0	PLB: 67.9 ± 8.2
Elfokery et al. 2018 [21], Egypt	Thoracotomy	3	Pneumonectomy (5), Lobectomy (25), Metastatectomy (70)	Incidence Severity of ISP Safety of PNB - PEFR - PaCO_2_	PNB: (*n* = 45) Periphrenic fat pad, 1–2 cm proximal at level of diaphragm, Bupivacaine SNB: (*n* = 45) Ultrasound guided (before operation), Bupivacaine	NO: (*n* = 45)	15.5 (PNB), 64.4 (SNB) vs. 66.7 (NO) (24 h)	PNB: 41 ± 7.8 SNB: 36.5 ± 7.2	NO: 39 ± 7.4
Krishnamoorthy et al. 2018 [16], United Kingdom	Thoracotomy	5	Lobectomy	Incidence Severity of ISP Safety of PNB - Peak flow volume	PNB: (*n* = 50) Perinephric fat pad above and below hilum of diaphragm, Bupivacaine	NO: (*n* = 50)	15.2 vs. 31.8	PNB: 74 ± 15	NO: 70 ± 13
Kuroiwa et al. 2020 [33], Japan	Thoracotomy (10.5), VATS (89.5)	3	Lobectomy (80), Wedge resection (17.6), Other (2.4)	Incidence Severity of ISP Safety - Range of shoulder movement - Nausea/Vomiting	PNB: (*n* = 42) (Rt) proximal to junction of azygous vein and SVC (Lt) hemivenous join the azygous vein, Ropivacaine	PLB: (*n* = 43) (Rt) proximal to junction of azygous vein and SVC (Lt) hemivenous join the azygous vein, 0.9% saline solution	33.3 vs. 46.5	PNB: 66.4 ± 10.8	PLB: 62.5 ± 18.5

PNB, phrenic nerve block; SNB, suprascapular nerve block; PLA, placebo; NR, not reported; NO, no intervention; Treat, treatment; Comp, comparator; VATS, video-assisted thoracoscopic surgery; FEV1, forced expiratory volume in 1 s; FVC, forced vital capacity; PEFR, peak expiratory flow rate; SVC, superior vena cava; Rt, right; LT, left.

**Table 2:** Comparisons of interventions for reduction in incidence of ISP during the first 24 h after surgery in network meta-analysis.

PNB		
0.43 (0.29,0.64)	**SNB**	
0.44 (0.34,0.58)	1.02 (0.71, 1.46)	**PLACEBO**

Data are expressed as RR (95% CI) of ISP in the column defining treatment compared with the row defining treatment. Column intervention compared with row intervention (SNB and placebo are reference compared to PNB). Significant results are in bold. RR = risk ratio; CI = confidence interval. PNB, phrenic nerve block; SNB, suprascapular nerve block.

**Table 3:** Comparisons of interventions for reduction of the severity of ISP during the first 24 h after surgery in network meta-analysis.

PNB		
−1.14 (−3.47, 1.19)	**SNB**	
−1.75 (−3.47, −0.04)	−0.61 (−2.95, 1.72)	**PLACEBO**

Data are expressed as WMD of ISP in the column define treatment compared with the row defining treatment. Column intervention compared with row intervention (SNB and placebo are reference compared to PNB). Significant results are in bold. Significant results are in bold. ISP, ipsilateral shoulder pain; WMD, weighted mean difference; CI, confidence interval; PNB, phrenic nerve block; SNB, suprascapular nerve block.

## Data Availability

Data will be shared upon reasonable request.

## Supplementary Materials

The following supporting information can be downloaded at https://www.mdpi.com/article/10.3390/medicina59020275/s1. Table S1. Search term details. Table S2. Description of intervention and cointervention of included studies. Table S3. Description of outcome measures of enrolled studies in network analysis. Table S4. Assessment of global inconsistency in networks using the “design-by-treatment” interaction model. Table S5. Assessment of loop inconsistency in networks. Table S6. Results of pairwise meta-analyses. Table S7. Results of sensitivity and subgroup analysis of network meta-analysis for prevention of ISP. Table S8. Results of sensitivity and subgroup analysis of network meta-analysis for reduction in ISP severity. Figure S1. Predictive interval plot of anesthetic intervention network for the incidence of ISP during 24 h after thoracic surgery. Figure S2. Network map of included studies for the severity of ISP. Figure S3. SUCRA ranking curves for reduction in ISP severity during the first 24 h after surgery. Figure S4. Network forest plot for reduction in ISP severity of each study during 24 h after surgery. Figure S5. Predictive interval plot of anesthetic interventions network for severity of ISP during 24 h after thoracic surgery. Figure S6. Comparison-adjusted funnel plot for the network of prevention of ISP in all comparisons. Figure S7. Comparison-adjusted funnel plot for the network of reduction of ISP severity in all comparisons.

## References

[1] Scawn N.D., Pennefather S.H., Soorae A., Wang J.Y., Russell G.N. (2001). Ipsilateral shoulder pain after thoracotomy with epidural analgesia: The influence of phrenic nerve infiltration with lidocaine. Anesth. Analg..

[2] Bunchungmongkol N., Pipanmekaporn T., Paiboonworachat S., Saeteng S., Tantraworasin A. (2014). Incidence and risk factors associated with ipsilateral shoulder pain after thoracic surgery. J. Cardiothorac. Vasc. Anesth..

[3] Barak M., Iaroshevski D., Poppa E., Ben-Nun A., Katz Y. (2007). Low-volume interscalene brachial plexus block for post-thoracotomy shoulder pain. J. Cardiothorac. Vasc. Anesth..

[4] Barak M., Zise A., Katz Y. (2004). Thoracic epidural local anesthetics are ineffective in alleviating post-thoracotomy ipsilateral shoulder pain. J. Cardiothorac. Vasc. Anesth..

[5] Danelli G., Berti M., Casati A., Bobbio A., Ghisi D., Mele R., Rossini E., Fanelli G. (2007). Ipsilateral shoulder pain after thoracotomy surgery: A prospective, randomized, double-blind, placebo-controlled evaluation of the efficacy of infiltrating the phrenic nerve with 0.2%wt/vol ropivacaine. Eur. J. Anaesthesiol..

[6] Martinez-Barenys C., Busquets J., de Castro P.E., Garcia-Guasch R., Perez J., Fernandez E., Mesa M.A., Astudillo J. (2011). Randomized double-blind comparison of phrenic nerve infiltration and suprascapular nerve block for ipsilateral shoulder pain after thoracic surgery. Eur. J. Cardiothorac. Surg..

[7] Pennefather S.H., Akrofi M.E., Kendall J.B., Russell G.N., Scawn N.D. (2005). Double-blind comparison of intrapleural saline and 0.25% bupivacaine for ipsilateral shoulder pain after thoracotomy in patients receiving thoracic epidural analgesia. Br. J. Anaesth..

[8] Saha S., Brish E.L., Lowry A.M., Boddu K. (2011). In select patients, ipsilateral post-thoracotomy shoulder pain relieved by suprascapular nerve block. Am. J. Ther..

[9] Tan N., Agnew N.M., Scawn N.D., Pennefather S.H., Chester M., Russell G.N. (2002). Suprascapular nerve block for ipsilateral shoulder pain after thoracotomy with thoracic epidural analgesia: A double-blind comparison of 0.5% bupivacaine and 0.9% saline. Anesth. Analg..

[10] Pipanmekaporn T., Punjasawadwong Y., Charuluxananan S., Lapisatepun W., Bunburaphong P., Boonsri S. (2018). The effectiveness of intravenous parecoxib on the incidence of ipsilateral shoulder pain after thoracotomy: A randomized, double-blind, placebo-controlled trial. J. Cardiothorac. Vasc. Anesth..

[11] Mac T.B., Girard F., Chouinard P., Boudreault D., Lafontaine E.R., Ruel M., Ferraro P. (2005). Acetaminophen decreases early post-thoracotomy ipsilateral shoulder pain in patients with thoracic epidural analgesia: A double-blind placebo-controlled study. J. Cardiothorac. Vasc. Anesth..

[12] Hodge A., Rapchuk I.L., Gurunathan U. (2021). Postoperative pain management and the incidence of ipsilateral shoulder pain after thoracic surgery at an Australian tertiary-care hospital: A prospective audit. J. Cardiothorac. Vasc. Anesth..

[13] Manzoor S., Khan T., Zahoor S.A., Wani S.Q., Rather J.M., Yaqoob S., Ali Z., Hakeem Z.A., Dar B.A. (2019). Post-thoracotomy ipsilateral shoulder pain: What should be preferred to optimize it–phrenic nerve infiltration or paracetamol infusion?. Ann. Card. Anaesth..

[14] Burgess F.W., Anderson D.M., Colonna D., Sborov M.J., Cavanangh D.G. (1993). Ipsilateral shoulder pain following thoracic surgery. Anesthesiology.

[15] Li W.W., Lee T.W., Yim A.P. (2004). Shoulder function after thoracic surgery. Thorac. Surg. Clin..

[16] Krishnamoorthy B., Critchley W.R., Soon S.Y., Birla R., Begum Z., Nair J., Devan N., Mohan R., Fildes J., Morris J. (2019). A randomized study comparing the incidence of postoperative pain after phrenic nerve infiltration vs. nonphrenic nerve infiltration during thoracotomy. Semin. Thorac. Cardiovasc. Surg..

[17] Mark J.B., Brodsky J.B. (1993). Ipsilateral shoulder pain following thoracic operations. Anesthesiology.

[18] Bamgbade O.A., Dorje P., Adhikary G.S. (2007). The dual etiology of ipsilateral shoulder pain after thoracic surgery. J. Clin. Anesth..

[19] Feray S., Lubach J., Joshi G.P., Bonnet F., Van de Velde M., the PROSPECT Working Group of the European Society of Regional Anaesthesia and Pain Therapy (2022). PROSPECT guidelines for video-assisted thoracoscopic surgery: A systematic review and procedure-specific postoperative pain management recommendations. Anaesthesia.

[20] The European Society of Regional Anesthesia and Pain Therapy Procedure Specific Postoperative Pain Management. https://esraeurope.org/prospect/procedures/thoracotomy-2015/intra-operative-7.

[21] Elfokery B.M., Tawfic S.A., Abdelrahman A.M., Abbas D.N., Abdelghaffar I.M. (2018). Comparative study on the analgesic effect of acute ipsilateral shoulder pain after open thoracotomy between preoperative ultrasound guided suprascapular nerve block (SNB) and intraoperative phrenic nerve infiltration (PNI) in cancer lung patients. J. Egypt. Natl. Canc. Inst..

[22] Blichfeldt-Eckhardt M.R., Laursen C.B., Berg H., Holm J.H., Hansen L.N., Ørding H., Andersen C., Licht P.B., Toft P. (2016). A randomised, controlled, double-blind trial of ultrasound-guided phrenic nerve block to prevent shoulder pain after thoracic surgery. Anaesthesia.

[23] Shanahan E.M., Ahern M., Smith M., Wetherall M., Bresnihan B., FitzGerald O. (2003). Suprascapular nerve block (using bupivacaine and methylprednisolone acetate) in chronic shoulder pain. Ann. Rheum. Dis..

[24] Ozyuvaci E., Akyol O., Sitilci T., Dübüs T., Oglu H.T., Leblebici H., Ikgöz A.A. (2013). Preoperatıve ultrasound-guided suprascapular nerve block for postthoracotomy shoulder paın. Curr. Ther. Res. Clin. Exp..

[25] Shim S., Yoon B.H., Shin I.S., Bae J.M. (2017). Network meta-analysis: Application and practice using Stata. Epidemiol. Health..

[26] Salanti G., Higgins J.P., Ades A.E., Ioannidis J.P. (2008). Evaluation of networks of randomized trials. Stat. Methods. Med. Res..

[27] Liberati A., Altman D.G., Tetzlaff J., Mulrow C., Peter C., Gøtzsche P.C., Ioannidis A.J.P., Clarke M., Devereaux P.J., Kleijnen J. (2009). The PRISMA statement for reporting systematic reviews and meta-analyses of studies that evaluate healthcare interventions: Explanation and elaboration. BMJ.

[28] Pipanmekaporn T., Leurcharusmee P., Punjasawadwong Y., Khorana J., Samerchua A., Sukhupragarn W., Sukuam I., Bunchungmongkol N., Saokaew S. (2020). Comparative Effectiveness of Phrenic Nerve Block and Suprascapular Nerve Blockfor Amelioration of Ipsilateral Shoulder Pain after Open Thoracotomy: A Systematic Review and Network Meta-Analysis. https://www.crd.york.ac.uk/prospero/display_record.php?RecordID=204473.

[29] Higgins J.P.T., Jacqueline C., Miranda C., Tianjing L., Matthew J.P., Vivian A.W. (2019). Cochrane Handbooks for Systematic Review of Interventions.

[30] Jadad A.R., Moore R.A., Carroll D., Jenkinson C., Reynolds D.J., Gavaghan D.J., McQuay H.J. (1996). Assessing the quality of reports of randomized clinical trials: Is blinding necessary?. Control. Clin. Trials..

[31] Jo J.K., Autorino R., Chung J.H., Kim K.S., Lee J.W., Baek E.J., Lee S.W. (2013). Randomized controlled trials in endourology: A quality assessment. J. Endourol..

[32] Hozo S.P., Djulbegovic B., Hozo I. (2005). Estimating the mean and variance from the median, range, and the size of a sample. BMC Med. Res. Methodol..

[33] Kuroiwa K.K., Shiko Y., Kawasaki Y., Aoki Y., Nishizawa M., Ide S., Miura K., Kobayashi N., Sehmbi H. (2021). Phrenic nerve block at the azygos vein level versus sham block for ipsilateral shoulder pain after video-assisted thoracoscopic surgery: A randomized controlled trial. Anesth. Analg..

[34] Morélot-Panzini C., Le Pimpec-Barthes F., Menegaux F., Gonzalez-Bermejo J., Similowski T. (2015). Referred shoulder pain (C4 dermatome) can adversely impact diaphragm pacing with intramuscular electrodes. Eur. Respir. J..

[35] Ohmori A., Iranami H., Fujii K., Yamazaki A., Doko Y. (2013). Myofascial involvement of supra- and infraspinatus muscles contributes to ipsilateral shoulder pain after muscle-sparing thoracotomy and video-assisted thoracic surgery. J. Cardiothorac. Vasc. Anesth..

[36] Blichfeldt-Eckhardt M.R., Andersen C., Ørding H., Licht P.B., Toft P. (2017). Shoulder pain after thoracic surgery: Type and time course, a prospective cohort study. J. Cardiothorac. Vasc. Anesth..

[37] Hung Y.A., Sun C.K., Chiang M.H., Chen J.Y., Ko C.C., Chen C.C., Chen Y., Teng I.C., Hung K.C. (2022). Effect of intraoperative phrenic nerve infiltration on postoperative ipsilateral shoulder pain after thoracic surgeries: A systematic review and meta-analysis of randomized controlled studies. J. Cardiothorac. Vasc. Anesth..

[38] Woo J.H., Kim Y.J., Kim K.C., Kim C.H., Jun J. (2018). The effect of interscalene block on ipsilateral shoulder pain and pulmonary function in patients undergoing lung lobectomy: A randomized controlled trial. Medicine.

[39] Blichfeldt-Eckhardt M.R., Toft P. (2019). Treatment of ipsilateral shoulder pain after thoracic surgery-time for comparative studies?. J. Thorac. Dis..

[40] Misiołek H., Karpe J., Copik M., Marcinkowski A., Jastrzębska A., Szelka A., Czarnożycka A., Długaszek M. (2014). Ipsilateral shoulder pain after thoracic surgery procedures under general and regional anesthesia–a retrospective observational study. Kardiochir. Torakochirurgia. Pol..

