# Supplementary material for: Efficacy of Phrenic Nerve Block and Suprascapular Nerve Block in Amelioration of Ipsilateral Shoulder Pain after Thoracic Surgery: A Systematic Review and Network Meta-Analysis
Source: Medicina (Kaunas). 2023 Jan 31;59(2):275. doi: 10.3390/medicina59020275 (PMC9962566; doi:10.3390/medicina59020275)
Supplement: Supplementary file 1 [file medicina-59-00275-s001.zip › medicina-2080285-supplementary.pdf]

Table S1. Search term details

Search Term	Database	Literature search		Search update	
		Citations	Date	Citations	Date
((ipsilateral) AND (shoulder pain)) AND (thorac*)) AND (surgery)	PubMed	62	9 November 2020	69	30 September 2022
TITLE-ABS ("shoulder pain" AND surgery AND (thoracic OR thoracotomy OR thoracoscopy)) AND (LIMIT-TO (DOCTYPE , "ar"))	Scopus	67	9 November 2020	75	30 September 2022
(ipsilateral and "shoulder pain" and thorac* and surgery).af.	Ovid database	33	9 November 2020	40	30 September 2022
ipsilateral AND shoulder pain AND thorac* AND surgery	Cochrane Library	34	9 November 2020	36	30 September 2022
ALL FIELDS: (ipsilateral) AND ALL FIELDS: (shoulder pain) AND ALL FIELDS: (thorac*) AND ALL FIELDS: (surgery)	Web of science	50	9 November 2020	50	30 September 2022
ipsilateral AND 'shoulder pain' AND thorac* AND surgery	EMBASE	56	9 November 2020	70	30 September 2022
allintitle: "shoulder pain" thoracotomy OR thoracoscopy	Google Scholar	37	9 November 2020	41	30 September 2022

Table S2. Description of intervention and co-intervention of included studies

ID	Authors	Intervention	Types and volume of local anesthetics		Co-interventions			Follow up period
			Type	Volume	TEA/TPVB	Rescue medication for ISP	Positioning	
1	Scawn et al. (2001)	PNB VS PLA	1%Lidocaine 10 ml	0.9% saline solution 10 ml	Continuous TEA at midthoracic level with bupivacaine and fentanyl	Ketorolac 10 mg IV for severe ISP	Upper arm flex < 90 degree to avoid excessive distraction of shoulder joint	6 h
2	Danelli et al. 2007	PNB vs PLA	0.2% Ropivacaine 10 ml	NSS 10 ml	Continuous TEA at T5-T6 or T6-T7 with ropivacaine	Paracetamol infusion 1 g every 8 h, subcutaneous morphine	Upper arm flex < 90 degree to avoid excessive distraction of shoulder joint	48 h
3	Martinez Barenys et al. (2011)	PNB VS SNB	2% Lidocaine 10 ml	0.5% Bupivacaine 10 ml	Continuous TEA at T5-T6 or T6-T7 interspace with bupivacaine and fentanyl	Dexketoprophen 50 mg IV every 8 h and metamizol 2 g IV	Upper arm flex < 90 degree to avoid excessive distraction of shoulder joint	72 h
4	Ozyuvaci et al. (2013)	SNB VS NO	0.5% levobupivacaine 10 ml	NO	Continuous TEA at T6-T7 interspace with Levobupivacaine and fentanyl	ND	ND	72 h
5	Blichfeldt-Eckhardt et al. (2016)	PNB VS PLA	1% Ropivacaine 10 ml and	NSS 10 ml and then 10 ml every 8	Continuous TEA at T6-T7 with	Paracetamol 1 g IV every 8 h, morphine IV,	ND	60 h

			then 10 ml every 8 h until intercostal drain removal or maximal of 3 days	hrs until intercostal drain removal or maximal of 3 days	bupivacaine + fentanyl	Ibuprofen or lornoxicam (Ibuprofen or lornoxicam was the first choice for treatment ISP)		
6	Elfokery et al. (2018)	PNB VS SNB VS PLA	PNB: 0.5% Bupivacaine 10 ml<br>SNB: 0.5% Bupivacaine 10 ml	NO	Continuous TEA at T6-7 interspace with bupivacaine and fentanyl	Ketorolac 30 mg IV over 50 ml saline for ISP and not exceed 120 mg/day	ND	48 h
7	Krishnam-oorthy et al. (2018)	PNB VS NO	PNB: 0.25% Bupivacaine 10 ml	NO	TPVB with bupivacaine at T5 and T8 levels with bupivacaine and epipleural catheter with continuous infusion with bupivacaine	PCA Morphine	ND	72 h
8	Kuroiwa et al. (2020)	PNB VS PLA	0.375% Ropivacaine 10 ml	0.9% saline solution 10 ml	Continuous TEA at T5-T6 or T6-T7 interspace with ropivacaine and fentanyl or morphine	Acetaminophen 1 gm IV	Upper arm was on an arm rest, fixed in a natural position	24 h

Abbreviations: NSS, normal saline; hrs, hours; TEA, thoracic epidural analgesia; TPVB, thoracic paravertebral nerve block; PNB, Phrenic nerve block; SNB, Suprascapular nerve block; PLA, placebo

Table S3. Description of outcome measures of enrolled studies in network analysis

Study	Outcomes
**Incidence of ISP**	
Scawn et al. (2001)	Ipsilateral shoulder pain (ISP) was assessed during 0.5, 1, 2, 3 ,4, 5 and 6 h after surgery. Patients were also asked to localize the pain area of the pain to differentiate shoulder pain from wound pain.
Danelli et al. (2007)	Presence of ISP at 6, 12, 24, 36 and 48 h after the end of surgery
Martinez Barenys et al. (2011)	ISP was assessed at 0.5, 1, 2, 3, 4, 5, 6, 12, 48 and 72 h after the operation and at discharge.
Blichfeldt-Eckhardt et al. (2016)	ISP was defined as any pain in the shoulder during the first six hour after the surgery
Elfokery et al. (2018)	ISP was assessed every 6 h for 48 h after the surgery.
Krishnamoorthy et al. (2018)	Preoperative pain score at shoulder was assessed. ISP was evaluated at 1, 3, 6,12,24,48, and 72 h after the surgical procedure during at rest and movement.
Kuroiwa et al. (2020)	The incidence of ISP was defined as the proportion of patients who reported a numeric rating scale (NRS) score more than 1 or equal at least once during 24 h after the operation.
**Severity of ISP**	
Scawn et al. (2001)	Degree of ISP was evaluated using a visual analogue scale (VAS) (0 mm = no pain and 100 mm = worst pain imaginable) and a 5-point verbal ranking score (VRS) (0- no pain, 1-mild pain, 2-moderate pain, 3-severe pain, 4-horrible pain) at 0.5, 1, 2, 3 ,4, 5 and 6 h after surgery.
Danelli et al. (2007)	The degree of ISP was assessed using a 100 mm VAS at 6, 12, 24, 36 and 48 h after the operation.
Martinez Barenys et al. (2011)	A-10 cm VAS (0 = no pain and 10 the worst imaginable pain score and a 5-point OVRS (no pain, slight pain, moderate pain, intense pain and intolerable) were used to measure shoulder pain severity at 0.5, 1, 2, 3, 4, 5, 6, 12, 48 and 72 h after the operation and at discharge.
Ozyuvaci et al. (2013)	The degree of ISP severity was measured using A-10 cm VAS before discharge from recovery room, during 1, 3, 6, 12, 24, 36, 48, and 72 h at rest and on coughing.
Blichfeldt-Eckhardt et al. (2016)	Intensity of ISP during the first three days. An 11 point-NRS was used to measure severity of shoulder pain during 6, 20, 28, 36, 44, 52 and 60 h after surgery. The pain scores recorded were represented the maximum ISP during the preceding time period.
Elfokery et al. (2018)	VAS of shoulder pain was assessed every 6 h after surgical procedure for 48 h.
Krishnamoorthy et al. (2018)	Severity of ISP was measured using a 5-point verbal ranking scale (0 = no pain, 1 = mild, 2= moderate, 3 = severe, 4 = unbearable) during at rest and movement at 3, 6,12,24,48, and 72 h after surgery.
Kuroiwa et al. (2020)	The degree of ISP at rest was measured using an 11-point NRS during 2, 4, 8,16,24 h after the operation.
**Respiratory function assessment**	
Scawn et al. (2001)	Baseline forced vital capacity (FVC) and forced expiratory volume in 1

	second (FEV1) were measured during preoperative period only
Danelli et al. (2007)	Preoperative ratio of FEV1/ FVC (%) were measured during preoperative period only.
Elfokery et al. (2018)	Peak expiratory flow rate (PEFR) was recorded at baseline and every 6 h postoperatively for 48 h when VAS was less than 3 or equal.
Blichfeldt-Eckhardt et al. (2016)	FEV1 and FVC at preoperative period and 2 h after operation were measured. The mean difference of FEV1 and FVC between preoperative and postoperative was presented.
Krishnamoorthy et al. (2018)	The peak flow measurement was obtained by using portable spirometer during preoperative period and postoperative period at 1 h, 3 h, 6 h, 12 h, 24 h, 48 h and 72 h.
**Ventilatory status by PaCO2**	
Scawn et al. (2001)	Blood gas analysis was measured during each assessment. Arterial carbon dioxide partial pressure ($PaCO_2$) during 0.5, 1, 2, 3 ,4, 5 and 6 h after surgery were shown.
Danelli et al. (2007)	Blood gas analysis was measured during preoperative period, 24 and 48 h after extubation. $PaCO_2$ and PaO_2/ FiO_2 ratio at baseline, the first and second postoperative days were presented.
Elfokery et al. (2018)	Arterial blood gas was assessed at baseline and every 6 h during postoperative period for 48 h. Partial pressure of arterial oxygen (PaO_2), $PaCO_2$, and arterial oxygen saturation were recorded.
Blichfeldt-Eckhardt et al. (2016)	Arterial blood gas was measured at 2 h after surgery. Partial pressure of arterial oxygen (PaO_2), $PaCO_2$, arterial pH was recorded.
**Upper Motor extremity weakness**	
Blichfeldt-Eckhardt et al. (2016)	The American Shoulder and Elbow Surgeons standardised shoulder strength assessment form was used to test motor blockade. Forward flexion, abduction, internal and external rotation were assessed by using a 6-point scale (0 = no contraction and 5 = normal power). Scores of 0-1 were defined as paralysis, 2-3 as severe paresis and 4 as mild paresis. The lowest score was recorded.
Kuroiwa et al. (2020)	Range of shoulder movement was assessed at baseline and postoperative period after removal of chest drain.

Table S4. Assessment of global inconsistency in networks using the 'design-by-treatment' interaction model

Network outcome	Chi-square	*p*-Value for test of global inconsistency
Incidence of ISP	3.30	0.192
Severity of ISP	42.65	< 0.001

Abbreviations: ISP, ipsilateral shoulder pain

Table S5. Assessment of loop inconsistency in networks

Closed triangular of quadratic loop of evidence	Inconsistency factor (95% CI)	*p*-Value	Loop heterogeneity tau²
**Incidence of ISP**			
PLACEBO- PNB-SNB	0.33 (0.00, 1.03)	0.345	0.015
**Severity of ISP**			
PLACEBO-PNB-SNB	3.97 (1.10, 6.84)	0.007	1.579

Abbreviations: ISP, ipsilateral shoulder pain; PNB, phrenic nerve block; SNB, suprascapular nerve block

Table S6. Results of pairwise meta-analyses

Comparisons			No of studies	Pairwise meta-analysis RR (95% CI)	Heterogeneity I^2
**Prevention of ISP**					
PNB	vs.	PLACEBO	5	0.48 (0.37 to 0.64)	0.0 %
SNB	vs.	PLACEBO	1	0.38 (0.27 to 0.54)	82.1%
PNB	vs.	SNB	2	0.35 (0.18 to 0.68)	52.3%
**Reduction severity of ISP**					
PNB	vs.	PLACEBO	6	-1.79 (-3.16 to -0.41)	88.2%
SNB	vs	PLACEBO	1	-1.00 (-2.11 to 0.11)	NA
PNB	vs.	SNB	2	-2.75 (-5.26 to -0.24)	97.0%
**Postoperative ventilatory status indicated by PaCO2**					
PNB	vs.	PLACEBO	3	0.25 (-2.71 to 3.20)	75.9%

Abbreviations: ISP, ipsilateral shoulder pain; PNB; Phrenic nerve block, SNB; Suprascapular nerve block, RR, risk ratio

Table S7. Results of sensitivity and subgroup analysis of network meta-analysis for prevention of ISP

**Interventions**	**Estimated effect of network meta-analysis; Risk ratio, RR (95% CI)**				
	Main analysis	Included ISP at 6 h after surgery	Included ISP at 24 h after surgery	Included studies with thoracotomy	Included only studies conducted since 2010
PNB*	**0.44 (0.34 to 0.58)**	**038 (0.27-0.53)**	**0.44 (0.29 to 0.68)**	**0.37 (0.27 to 0.51)**	**0.44 (0.29 to 0.65)**
SNB *	1.02 (0.71 to 1.46)	0.85 (0.56-1.28)	1.05 (0.62 to 0.77)	0.96 (0.72 to 1.26)	1.04 (0.63 to 1.73)
PNB#	**0.43 (0.29 to 0.64)**	**0.45 (0.30-0.68)**	**0.42 (0.24 to 0.72)**	**0.39 (0.27 to 0.55)**	**0.42 (0.25 to 0.70)**
Overall inconsistency Chi-square (*p*-Value)	0.192	0.067	0.218	0.318	0.293
Numbers of studies	7	7	5	5	5

* Results compared to placebo # Results compared to SNB

Abbreviations: ISP, ipsilateral shoulder pain, PNB, phrenic nerve block; SNB, suprascapular nerve block

Table S8. Results of sensitivity and subgroup analysis of network meta-analysis for reduction of ISP severity

Interventions	Estimated effect of network meta-analysis; Weighted Mean difference, MD (95% CI)					
	Main analysis	Included ISP at 6 hours after surgery	Included ISP at 24 hours after surgery	Included only studies with thoracotomy	Included only studies conducted since 2010	Included only high-quality studies
PNB*	**-1.75 (-3.47 to -0.04)**	**-2.02 (-3.21, -0.84)**	**-2.27 (-4.05 to -0.48)**	-2.22 (-4.70 to 0.27)	**-2.27 (-4.05 to -0.48)**	-1.47 (-3.17 to 0.22)
SNB *	- 0.61 (-2.95 to 1.72)	- 0.54 (-2.10, 1.01)	-0.87 (-3.10 to 1.36)	-0.85 (-3.65 to 1.95)	-0.87 (-3.10 to 1.36)	0.44 (-2.31 to 3.18)
PNB#	-1.14 (-3.47, to 1.19)	-1.48 (-3.05,0.08)	-1.40 (-3.63 to 0.83)	-1.37 (-4.17 to 1.44)	-1.40 (-3.63 to 0.83)	-1.91 (-4.42 to 0.60)
Overall inconsistency Chi-square (*p*-Value)	< 0.001	0.575	< 0.001	< 0.001	< 0.001	< 0.001
Numbers of studies	7	7	6	5	6	6

*Results compared to placebo # Results compared to SNB

Abbreviations: ISP, ipsilateral shoulder pain, PNB, phrenic nerve block; SNB, suprascapular nerve block

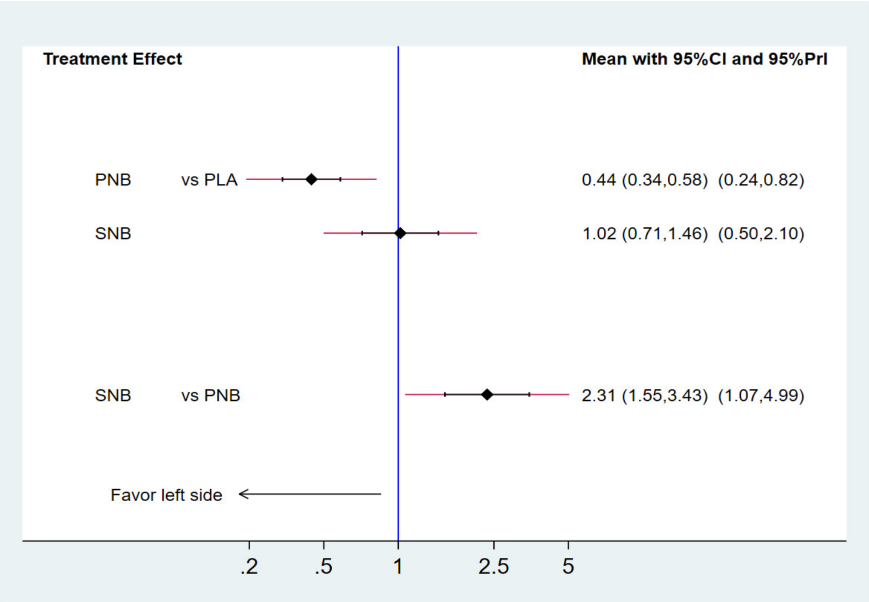

Figure S1. Predictive interval plot of anesthetic interventions network for the incidence of ISP during 24 h after thoracic surgery. The solid black lines represent the confidence intervals for summary risk ratio or mean difference for each comparison and the red lines the respective predictive intervals. The solid blue line corresponds to the line of no difference (RR = 1 or mean difference = 0). The arrow with "favor right or left" if the confidence interval is significant in the same direction of arrow. The anesthetic intervention in the designated direction (right/ left) is more favorable that the intervention in the other direction.

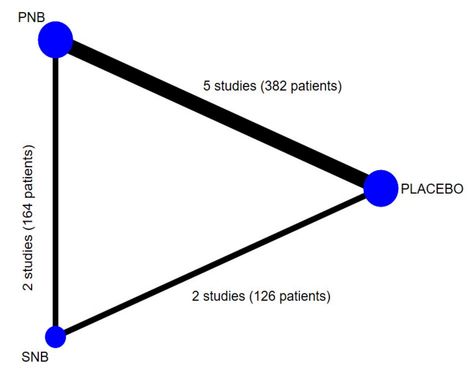

Figure S2. The network map of included studies for the severity of ISP. Width of the lines was the number of trials comparing pairwise intervention. Size of the circle was the proportion of sample size

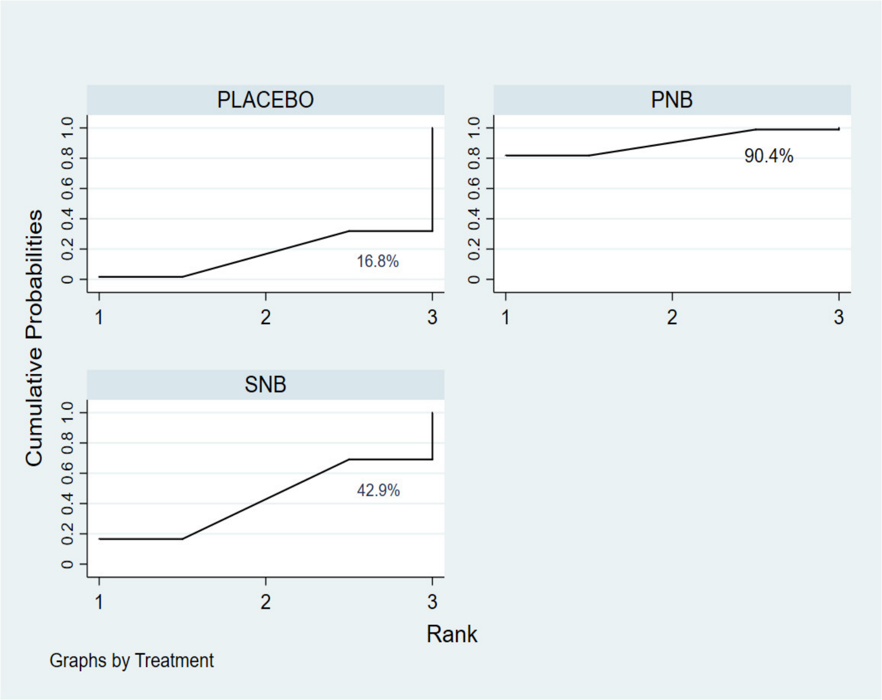

Figure S3. SUCRA ranking curves for reduction of ISP severity during the first 24 h after surgery

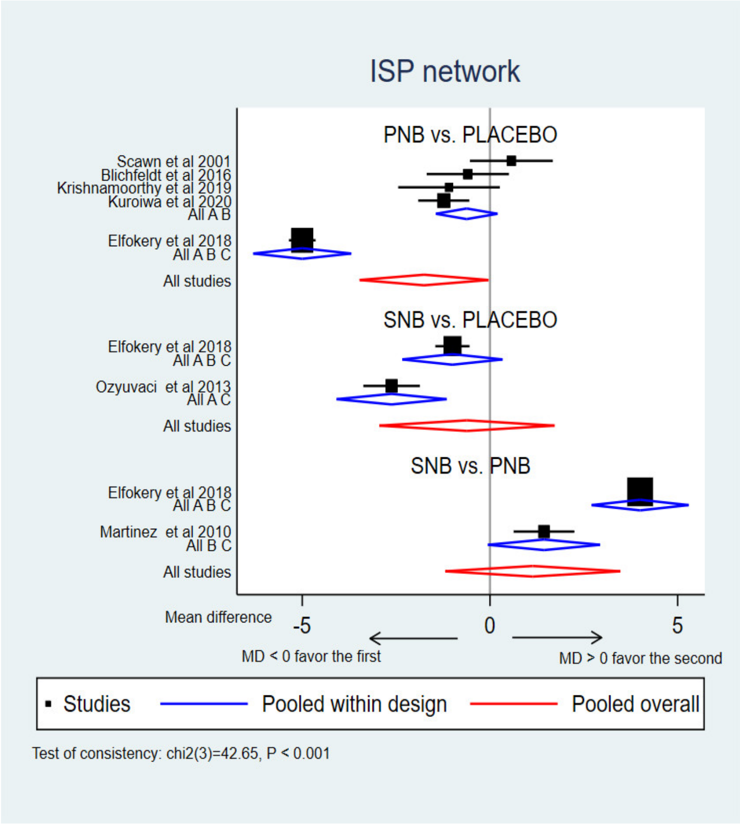

Figure S4. Network forest plot for reduction of ISP severity of each study during 24 h after surgery. The effect of PNB on ISP severity was comparable to SNB and placebo and the effect on SNB was also comparable to placebo. Forest plot: mean difference < 0 indicated that the first treatment was favored compared to the second. Mean difference > 0 indicated that the second treatment was favored compared to the first

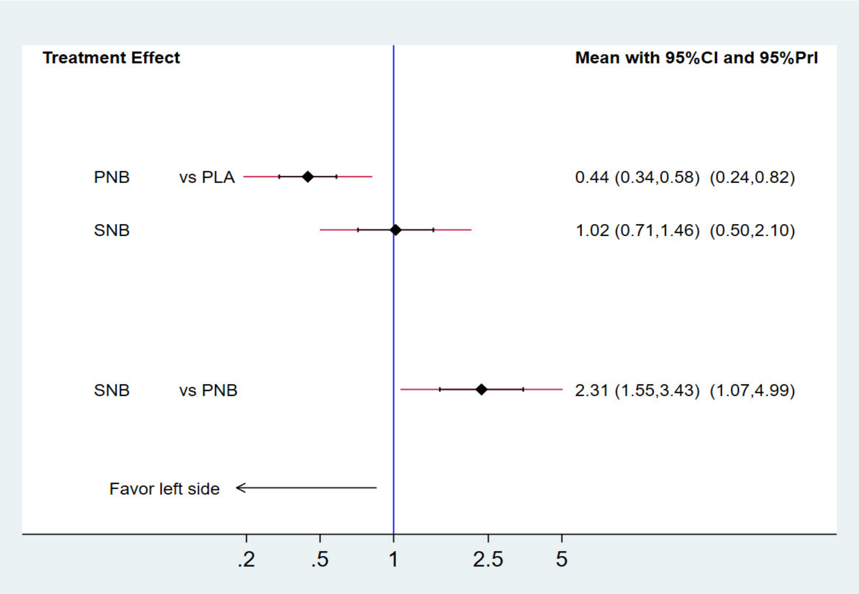

Figure S5. Predictive interval plot of anesthetic interventions network for severity of ISP during 24 hours after thoracic surgery. The solid black lines represent the confidence intervals for mean difference for each comparison and the red lines the respective predictive intervals. The solid blue line corresponds to the line of no difference (mean difference = 0). The arrow with "favor right or left" if the confidence interval is significant in the same direction of arrow. The anesthetic intervention in the designated direction (right/ left) is more favorable that the intervention in the other direction.

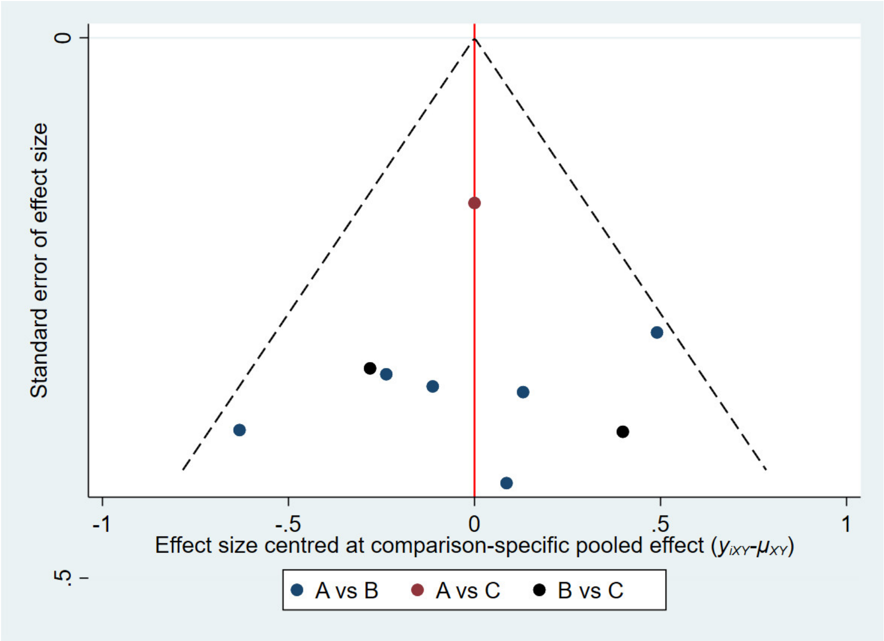

Figure S6. Comparison-adjusted funnel plot for the network of the prevention of ISP in all comparisons. A = PLACEBO, B = Phrenic nerve block, C = Suprascapular nerve block

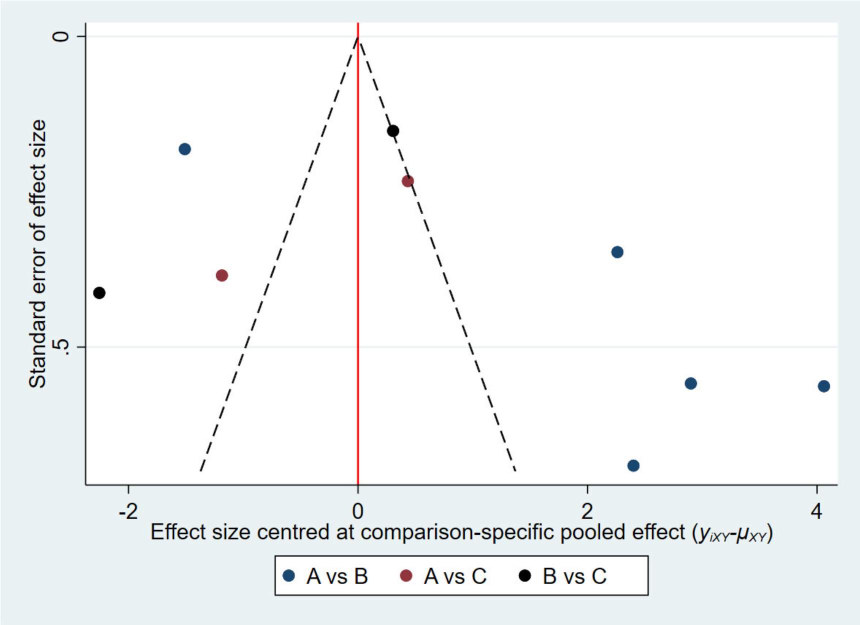

Figure S7. Comparison-adjusted funnel plot for the network of the reduction of ISP severity in all comparisons. A = PLACEBO, B = Phrenic nerve block, C = Suprascapular nerve block
